# Amino-Functionalized Multiwall Carbon Nanotubes as Efficient Basic Catalysts for the Formation of γ-Lactams: Synthesis of N-1-Heptenyl-2-pyrrolidinone

**DOI:** 10.3390/nano12040684

**Published:** 2022-02-18

**Authors:** Niurka Barrios-Bermúdez, Arisbel Cerpa-Naranjo, María Luisa Rojas-Cervantes

**Affiliations:** 1 Departamento de Química Inorgánica y Química Técnica, Facultad de Ciencias, UNED, Urbanización Monterrozas, Las Rozas, 28232 Madrid, Spain; niurka.barrios@universidadeuropea.es; 2 Departamento de Ciencias, Escuela de Ingeniería, Arquitectura y Diseño, Universidad Europea de Madrid, c/ Tajo s/n, Villaviciosa de Odón, 28670 Madrid, Spain; 3 Departamento de Ingeniería Industrial y Aeroespacial, Escuela de Ingeniería, Arquitectura y Diseño, Universidad Europea de Madrid, c/ Tajo s/n, Villaviciosa de Odón, 28670 Madrid, Spain; arisbel.cerpa@universidadeuropea.es

**Keywords:** amino-functionalized carbon nanotubes, basic catalysts, γ-lactams

## Abstract

In this work, we prepared a series of N-functionalized carbon nanotubes by means of a process of acylation-amidation of commercial multiwall carbon nanotubes that were previously pre-oxidized with nitric acid. Three different amines, butylamine, N,N-dimethyl ethylenediamine, and ethylenediamine, were used in the process. The characterization of samples by several techniques probed the incorporation of nitrogen atoms to the carbon nanotubes, especially in the case of ethylenediamine. The solids were tested as catalysts in the synthesis of N-1-heptenyl-2-pyrrolidinone, included in the group of a γ-lactams, compounds that show important biological properties. The most active catalyst was that prepared with butylamine, which exhibited the highest S_BET_ and V_pore_ values and contained an amount of nitrogen that was intermediate between that of the other two catalysts. A yield of 60% to N-1-heptenyl-2-pyrrolidinone was achieved after 3 h at 120 °C under free-solvent conditions. This catalyst could be used in four consecutive cycles without significant activity loss.

## 1. Introduction

Carbon nanotubes (CNTs) are exceptional materials showing excellent electronic, physical, and chemical properties [1,2,3], which find interesting applications in the field of catalysis [4,5,6] as alternative supports to the conventional ones due to their special characteristics, including high mesoporosity, controlled pore size distribution, and high surface-active-site-to-volume ratio.

The acidic/basic character of the surface of CNTs can be modified by the incorporation of different heteroatoms or functionalities. One of these heteroatoms is nitrogen, which can be introduced by synthesis of the carbon nanotubes in the presence of a source of nitrogen [7,8,9] or incorporated to the carbon nanotubes by the insertion of functional groups [10,11,12]. The nitrogen atoms behave as electron donors [13,14], conferring basic character to the surface of carbon nanotubes. 

The carbon–carbon or carbon–nitrogen bond formation reactions are of great interest in the field of high-added-value product synthesis. Among the first of these approaches, Knoevenagel condensation leading to the formation of interesting products in the field of fine chemicals, has been widely used for testing the surface basicity of solid base catalysts [15,16]. Regarding these basic catalysts, metal-free carbon materials are experiencing a continuous growth due to their advantages over heterogeneous metal-based systems, such as the absence of metal leaching, poisoning/passivation, and sintering. In this sense, nitrogen-doped carbon nanotubes have been studied in Knoevenagel transformation [17], the hydrogenation of nitrobenzene [12], and the aldol condensation of furfural and acetone [8,9], among other base-catalyzed reactions. Furthermore, different articles in the literature evidence the basic properties of other free metal carbonaceous materials, such as carbon nitrides [18,19] or carbon nanotubes functionalized with aziridine [20], which were also successfully tested in Knoevenagel condensation. 

Numerous organic containing carbon–nitrogen bonds are of great interest because of their biological or pharmacological activity. Among them, gamma-lactams rings are nitrogenous heterocycles of great interest as constituents of many natural and non-natural compounds that cover a very broad range of biological and medical applications, and they are very useful as versatile synthetic intermediates [21]. Their synthesis is carried out through several organic reactions starting from acyclic or cyclic precursors [22] or through the transformation of pre-existing lactams by means of C-C or C-heteroatom bond formation [23]. The isocyanide-based multicomponent reactions are another widely reported route for the synthesis of lactams [24]. However, the synthesis usually consists of multiple reaction steps in the presence of organic solvents, sometimes under strict reaction conditions [25,26] or catalyzed by expensive transition metal compounds [27]; therefore, it is necessary to develop alternative synthetic approaches that are more efficient for the preparation of γ-lactam structures. In this sense, in the present work, we report, for the first time, an efficient and eco-friendly alternative to obtain N-substituted gamma-lactams by means of the carbon–nitrogen coupling of 2-pyrrolidinone and 1-heptanal over carbon nanotubes functionalized with amine groups in solvent-free conditions and with only one reaction step. The catalysts were previously tested with success in the Knoevenagel condensation between benzaldehyde and two methylenic compounds.

## 2. Materials and Methods

### 2.1. Preparation of Samples

The multiwall carbon nanotubes (named as CNT herein) used as pristine material for the synthesis of the catalysts were provided by Sigma-Aldrich, with the following characteristics: OD × L 6–9 nm × 5 µm and purity > 95%. They were previously functionalized by oxidation with nitric acid, to obtain the CNT-O sample, according to the procedure described in [28]. The commercial carbon nanotubes used as raw material contained CoCu_2_Sn as an impurity. However, it was mostly removed after treatment with nitric acid [28]. Then, 500 mg of carboxylated multiwalled carbon nanotubes (CNT-O) were stirred in a mixture of 120 mL of thionyl chloride (SOCl_2_) and 3 mL of dimethylformamide (DMF) at 75 °C for 24 h. After the acyl chlorination, the nanotubes were filtered and washed with anhydrous tetrahydrofuran (THF) several times and dried at room temperature for 24 h. This sample was named as CNT-OCl. Then, 300 mg of CNT-OCl were refluxed with 10 mL of amine solution and 150 mL of DMF at 100 °C for 24 h. After cooling to room temperature, the sample was filtered, and washed in several portions with DMF (150 mL) and with ethanol (150 mL) to remove excess amine. Finally, the solid was dried at 70 °C overnight. The amines used in the functionalization were butylamine (primary amine), N,N-dimethyl ethylenediamine (primary and tertiary diamine), and ethylenediamine (primary diamine). The samples thus prepared were nominated as CNT-But, CNT-N,N, and CNT-Et, respectively. We refer to the amine functionalized samples as CNT-A.

### 2.2. Characterization of Samples

Elemental analysis of CNT-A samples was carried out in a Analizador Elemental LECO CHNS-932 (Leco Instrumentos S.L., Madrid, Spain).

The textural properties of samples were determined from the nitrogen adsorption–desorption isotherms at −196 °C, using the Micromeritics ASAP 2010 equipment (Micromeritics, Méringac, France.) The samples were previously outgassed at 150 °C for 8 h until a vacuum set point of 200 mHg. The surface area was calculated by the BET method and the mesoporous properties of the samples (mesopore size distribution, mesopore volume, and average mesopore diameter) were obtained by the BJH (Barrett, Joyner, and Halenda) method. 

The point of zero charge (PZC) was measured following a similar procedure to that described in [29] with some modifications. For more details, see the complete description in [30].

Thermal analysis (TG-DTA) of CNT-O and CNT-A samples was carried out using a TGA-DTA-DSC Q600 (Waters Cromatografía, TA Instruments, Barcelona, Spain) system. Samples of about 20 mg were heated in helium from 30 up to 1000 °C (flowrate = 100 mL/min) with a heating rate of 10 °C/min. Infrared spectra of CNT-O and CNT-A samples were registered in a FT-IR Nicolet iS50 (Thermo Scientific) instrument (Thermo Fisher Scientific, Madrid, Spain), equipped with ATR analyzer of germanium in the 4000–700 cm^−1^ range. X-ray diffraction patterns were recorded using a X’Pert Pro Panalytical diffractometer (Malvern Panalytical, B.V., San Sebastián de los Reyes, Madrid, Spain) with CuKα radiation (1.5406 Å), operating at 40 kV and 40 mA. 

The morphology of the samples was analysed by scanning electron microscopy (SEM) using a JEOL JSM 6335F microscope (JEOL, Austin, TX, USA) operating at 200 kV, which also performed energy-dispersive X-ray spectroscopy (EDX) measurements. Transmission electron microscopy (TEM) images were registered using an Oxford Instrument, model X-Max (Oxford Instruments Nanoanalysis &Asylum Research, HighWycombe, UK) of 80 mm^2^ and with a resolution between 0.127 and 5.9 KeV.

### 2.3. Reaction Procedure

Knoevenagel condensation between benzaldehyde and ethyl cyanoacetate was carried out under inert atmosphere in the absence of a solvent by mixing equimolar amounts of both reactants (14 mmol of each) in a three-necked reactor on a StarFish multi-experiment workstation, equipped with thermometer. After equilibrating the reactants at 90 °C, the catalyst (1 wt.%) was added. The samples, taken periodically from the batch reactor at selected reaction times, were filtered to remove the catalyst and analysed by gas chromatography in an Agilent 6890 GC device (Agilent Technologies, Santa Clara, CA, USA). The conversion was expressed in terms of the amount of benzaldehyde (in wt.%) transformed. Knoevenagel condensation between benzaldehyde and ethyl acetoacetate was carried out following the same procedure but using 9 mmol of each reactant and 2 wt.% of catalyst, with 120 °C as the reaction temperature.

To carry out the reaction to form N-substituted-gamma-lactam, the gamma-lactam 2-pyrrolidinone (3 mmol) and the catalyst (50 mg) were blended in a three-necked reactor on a StarFish multi-experiment workstation and equilibrated at 120 °C. Then, 9 mmol of 1-heptanal were added in the absence of any solvent. The samples were taken and analysed as explained above. The mass spectra of the products were obtained on a Hewlett-Packard HP5971A spectrometer (Scientific Instrument Services, Palmer, MA, USA). The conversion was expressed in terms of the amount of 2-pyrrolidinone (in wt.%) transformed.

Some experiments focused on the recyclability of the catalysts were carried out. After each reaction, the catalysts were filtered, washed with distilled water, and dried at 150 °C for 3 h in an oven. Due to the loss of catalyst produced between successive cycles, the amounts of reactants were rescaled for each cycle according to the amount of catalyst. Furthermore, only one sample was extracted and analysed during the reaction, at the time of 120 min, from the second cycle onwards. 

## 3. Results and Discussion

### 3.1. Results of Characterization

The results of the elemental analysis of the samples are given in Table 1, together with the corresponding nitrogen contents expressed in terms of mmol amine/g CNT.

The content of amine followed the order CNT-N,N < CNT-But < CNT-Et. Therefore, the sample containing the highest amount was that which was treated with ethylenediamine. This result was expected considering that, for this diamine, two nitrogen atoms could be incorporated to the carbon nanotubes, either with both anchored to the C=O groups, or with only one anchored to the group and the other remaining as -NH_2_ linked to the -CH_2_ groups of the amine structure. A slightly lower degree of functionalization was achieved for the CNT-But sample, in which a primary amine with a longer chain length was incorporated. Finally, in the case of N,N-dimethyl ethylenediamine, there was only a possibility of anchoring to the walls of carbon nanotubes, through the terminal -NH_2_ group, and the steric hindrance of methyl groups led to the lowest incorporation of the amine. Therefore, it seems that the longer the chain amine is and the more sterically hindered the nitrogen atom is, the lower the functionalization degree will be.

**Figure 1:**
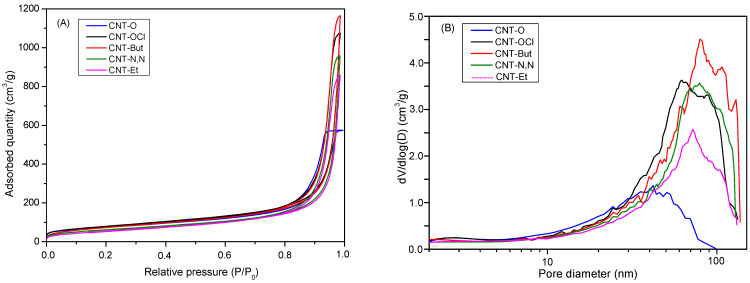
(**A**) Isotherms and (**B**) BJH distribution (adsorption branch) curves for CNT-O, CNT-OCl, and CNT-A samples.

As deduced from the isotherm shapes (Figure 1A) and the volumes of micro- and mesopores (Table 2), the samples were mesoporous solids, mainly due to the voids existing between the bundles. When the oxidized nanotubes were treated with the acyl chloride, an increment in the V_meso_ and S_BET_ occurred (Table 2), as well as a shift in the BJH curve towards a bigger diameter (Figure 1B). This means that the acylation treatment led to a higher separation between the bundles. The incorporation of the amines resulted in a diminishing in the S_BET_ and V_pore_, due probably to the blockage of the pores by the aliphatic chains of the amines. This decrease was more remarkable in the case of CNT-Et sample, for which the amount of incorporated amine (as determined by elemental analysis) was higher. The shape of the BJH curves for CNT-A samples was not significantly altered with respect to that for CNT-OCl; however, for CNT-But and CNT-N,N, the average mesopore diameter was higher (Table 2 and Figure 1B).

The PZC value of pristine CNT was 6.4. When oxidation occurred, the character of the surface of carbon nanotubes changed from almost neutral to acidic (PZC of 3.4 for CNT-O, Table 2). The anchoring of the amines to the surface was corroborated by its slightly basic character, which resulted in PZC values close to 8.

**Figure 2:**
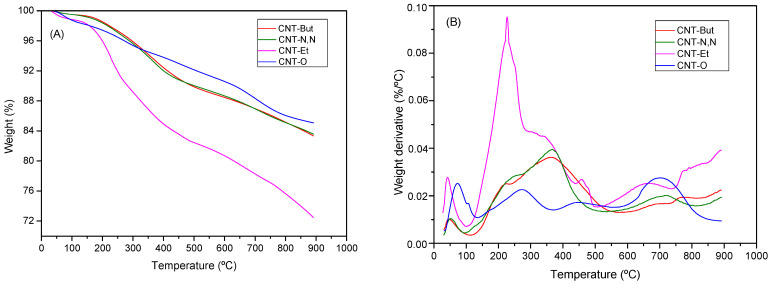
(**A**) TG and (**B**) DTG curves in helium for CNT-O and CNT-A samples.

The TG and DTG curves of CNT-O and CNT-A samples in helium are displayed in Figure 2. Several losses were observed in the TG curve of CNT-O. The first step, until 130 °C, corresponds to the loss of adsorbed water on the hydrophilic surface of oxidized carbon nanotubes [31,32]. The weight loss in the 150–350 °C range, with a maximum in the rate decomposition around 275 °C, can be assigned to the decarboxylation of the carboxylic groups [32]. The removal of the hydroxyl functionalities attached to the walls of carbon nanotubes is known to occur in the 350–550 °C interval [31]. Finally, the weight loss occurring above 600 °C can be attributed to the thermal oxidation of the remaining disordered carbon [33].

It can be seen (Figure 2A) that the weight loss for the functionalized samples was higher than for the oxidized one, indicating that the incorporation of amines to the structure of carbon nanotubes had been produced, especially in the case of ethylenediamine, which had been incorporated to a greater extent (weight loss of 27.6%, as compared to 16.6% for the other two amine samples). As a result, the DTG curve for CNT-Et (Figure 2B) exhibited two maxima in the 150–500 °C range, the intensities of which were significantly higher than those of the peaks detected in the DTG curves for the other CNT-A samples. The weight losses observed in the 150–600 °C range could be transformed into mmol/g CNT to obtain the degree of functionalization (Table 3), resulting in similar values to those obtained by elemental analysis (Table 1), except for CNT-Et. 

**Figure 3:**
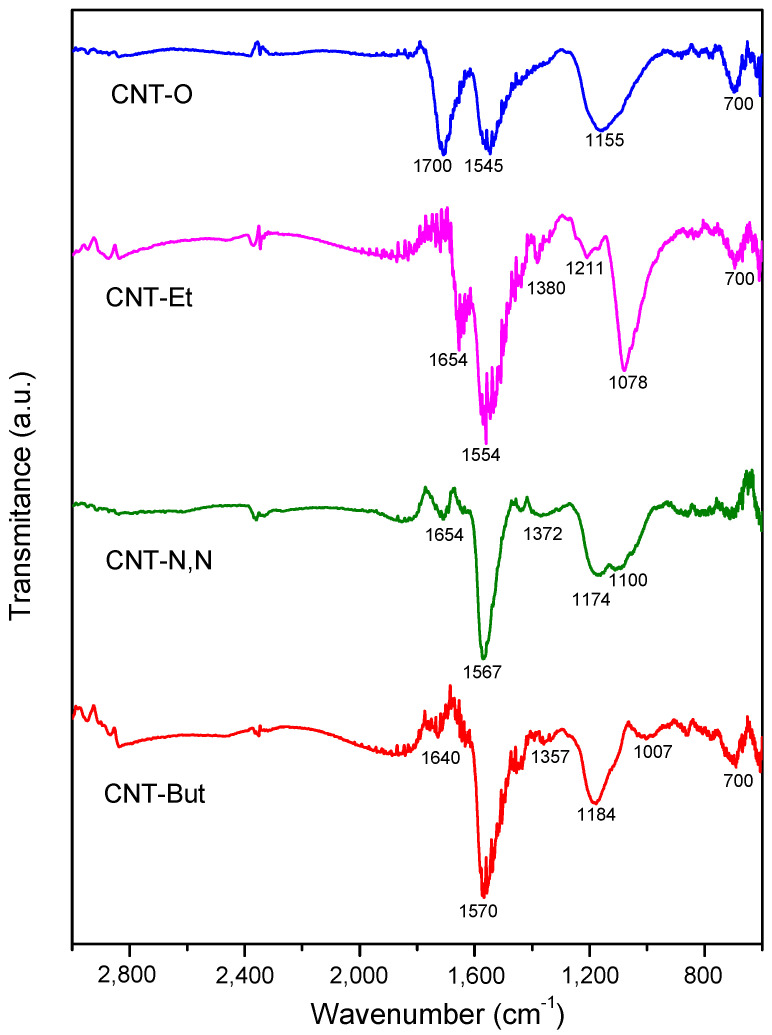
FTIR spectra for CNT-O, CNT-But, CNT-N,N, and CNT-Et samples.

The ATR-FTIR spectra of samples are represented in Figure 3. The relative intensity of the band placed at 1700 cm^−1^, assigned to C=O stretching vibration in carboxylic groups [34,35], with respect to that located at 1545 cm^−1^ (associated to -C≡C- vibrations of skeleton of carbon nanotubes) [34,36] decreased significantly for the amine-functionalized samples, as compared to the oxidized carbon nanotubes. This implies a decrease in the number of carboxylic groups, which were transformed into amide groups, and a new band at 1640–1654 cm^−1^ assigned to the stretching vibrations of C=O in the amide groups appeared [37,38]. This band appeared as a shoulder in the case of CNT-But and CNT-N,N, and with high intensity in the spectrum of CNT-Et, indicating the presence of a higher number of amide groups for this last sample. In addition, the band at 1545 cm^−1^ shifted to higher wavenumbers (1554–1570 nm) in the CNT-A samples, due to the stretching vibrations of the C-N bonds [37]. The band placed at 1175 cm^−1^ in the spectrum of CNT-O, associated with the C-O stretching vibrations of carboxylic groups [36,39], was clearly shifted to 1078 cm^−1^ in the CNT-Et spectrum (a band of low intensity at 1100 cm^−1^ was observed for CNT-But and CNT-N,N). The higher anchoring of the ethylenediamine with respect to the other two amines was also corroborated by the presence of the band placed at 2870 cm^−1^, due to the C-H stretching vibrations of the attachment of the -CH_2_- groups [35,40]. In summary, the ATR-FTIR spectra of sample probes show that the anchoring of the amines to the structure of carbon nanotubes had been produced, and this occurred to a greater extent in the case of ethylenediamine, as also detected by TG-DTG analysis.

**Figure 4:**
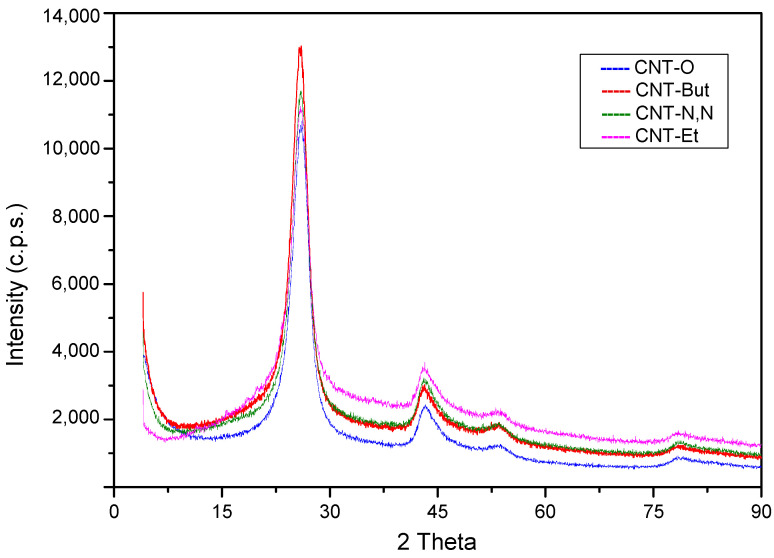
X-ray diffraction patterns for CNT-O, CNT-But, CNT-N,N, and CNT-Et samples.

From Figure 4, it can be deduced that functionalization with amines did not alter the general structure of CNTs, because the XRD patterns of amine functionalized CNTs were similar those of CNT-O. Furthermore, the crystallite size of graphite determined by the Scherrer equation was similar in all cases, between 3.2 and 3.4 nm. This means that the cylinder wall structure and the interplanar spacing remained the same. 

**Figure 5:**
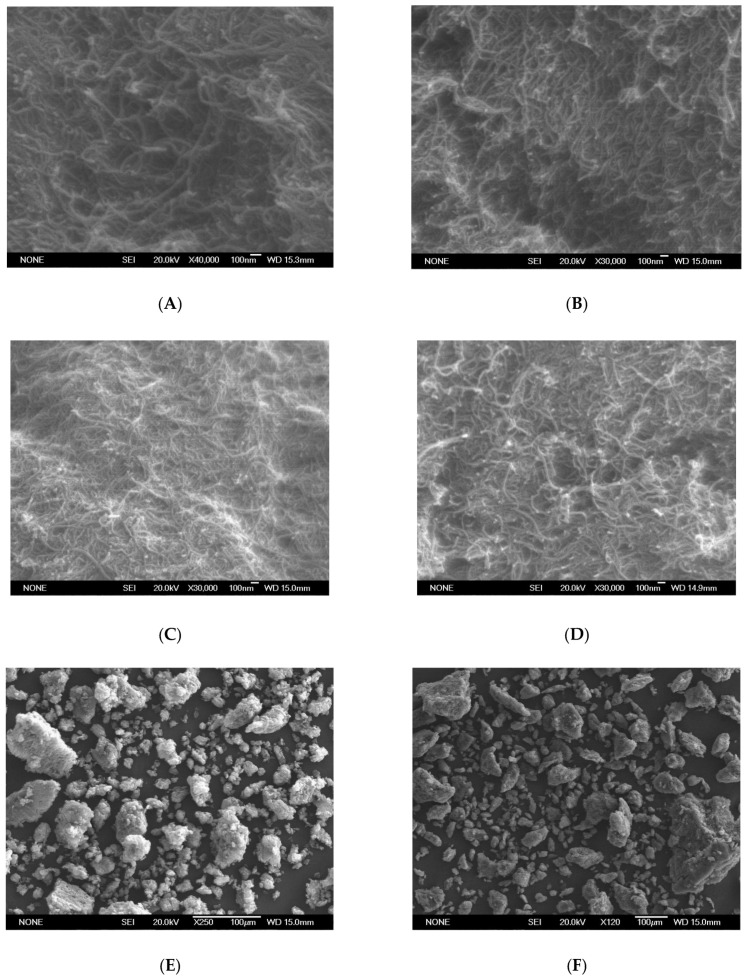
Selected SEM images of (**A**) CNT-OCl; (**B**) CNT-But; (**C**) CNT-N,N; (**D**) CNT-Et; (**E**) CNT-OCl; (**F**) CNT-Et.

The SEM images of carbon nanotubes that were acylated and functionalized with amines are displayed in Figure 5. The tubular structures of the nanotubes and their disposition in bundles was preserved in the CNT-A samples. Additionally, the anchoring of amines resulted in some brilliant zones in the corresponding spectra, especially in the case of ethylenediamine, where white dots of cotton-like features, mainly congregating on the tips on carbon nanotubes, were observed (Figure 5D). When comparing Figure 5E,F, it can be seen that the size of particulate aggregates of carbon nanotubes was higher for CNT-OCl (125–180 µm, some of 330 µm) than for CNT-Et (40–80 µm, some of 100 µm). This could be due to the steric hindrance of the anchoring alkyl chains, which prevented the grouping of bundles.

**Figure 6:**
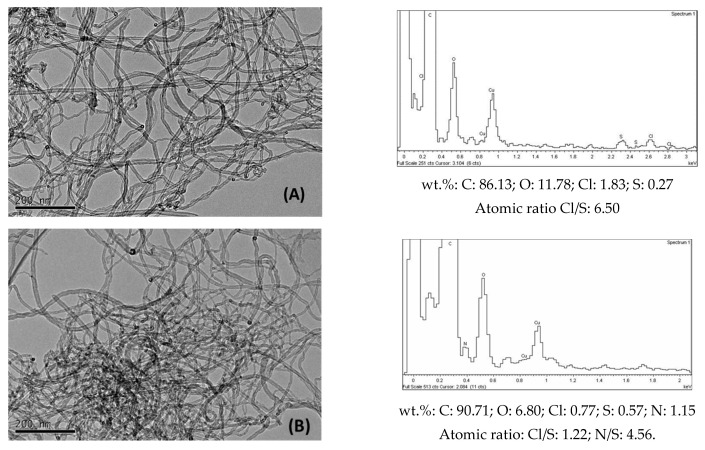

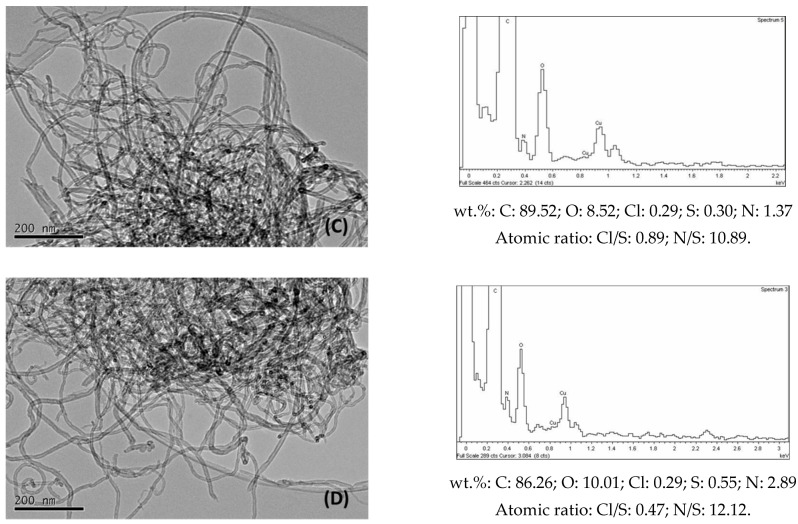
Selected TEM images and corresponding EDX spectra of (**A**) CNT-OCl; (**B**) CNT-But; (**C**) CNT-N,N; (**D**) CNT-Et.

The TEM images of functionalized samples (Figure 6) show darker zones due to the presence of amines. In the EDX spectrum of CNT-OCl, sulphur was unexpectedly found to be present, similarly to that found by Tesonier et al. [41]. It may have been the case that during the acylation step with SOCl_2_, additional reactions could take place, resulting in the adsorption or anchoring of sulphur-containing groups. The presence of nitrogen in all the functionalized samples was confirmed by the corresponding EDX spectra (Figure 6), where the amounts of elements in wt.% were also indicated. When the nitrogen amounts were transformed from wt.% into mmol amine/g CNT, the obtained values (Table 3) were lower than the values corresponding to those obtained by elemental analysis (Table 1). It must be considered that this last technique involves the bulk of sample, in contrast to the EDX measurements, which are carried out in specific points at the surface of samples.

The absorption technique in the UV-vis region is useful for studying the colloidal stability of nanotubes in suspension [42,43], as well as the possible disruption of the structure of nanotubes by covalent functionalization. With the purpose of checking the anchoring of the amines to the carbon nanotubes, we registered the absorption spectra for CNT-O and CNT-A samples.

**Figure 7:**
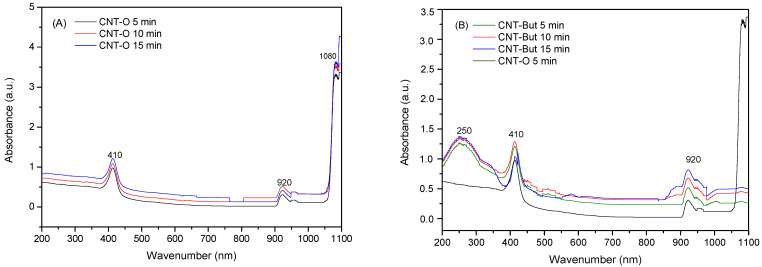
UV-Vis spectra for (**A**) CNT-O sonicated at different times; (**B**) CNT-O sonicated at 5 min and CNT-But sonicated at several time-points.

Figure 7A shows the absorption spectra for oxidized carbon nanotubes, sonicated at different times. A peak was observed at around 410 nm, which was attributed to the presence of a high number of benzene rings [44,45], and particularly to the existence of multi-walled nanotubes [46], in which transitions π→π* occurred. Additionally, two peaks centred at 920 nm and 1080 nm were detected. These peaks were due to Van Hove singularities in the density of states (DOS) and they corresponded with the band gap transitions in semiconducting nanotubes [47,48]. As observed in Figure 7A, the dispersion of nanotubes, and therefore, the absorbance, increased with the sonication time [49].

The UV-Vis spectra for CNT-But, as an example of CNT-A samples, are depicted in Figure 7B. Regarding the spectrum of the oxidized nanotubes, the disappearance of the van Hove singularity located at 1080 nm was observed. This may have been due to a partial covalent functionalization of the nanotubes with the amide groups [50], causing the electronic perturbation of the nanotubes and a disruption on the extended π network [50,51], although the singularity located at 920 nm remained. A new band was detected, centred around 250 nm, which can be attributed to the presence of NH groups from the amide functionalities [44]. The functionalization with the amine increased the dispersion of the carbon nanotubes, as deduced by the increment in absorbance units of 0.2 in the baseline of the curve for CNT-But (5 min) with respect to that for CNT-O (5 min). The spectra for the other two CNT-A samples were similar to that for CNT-But and, for simplicity, they are not shown. In conclusion, the results obtained by this technique also confirm the anchoring of the amines to the carbon nanotubes.

### 3.2. Catalytic Activity

**Figure 8:**
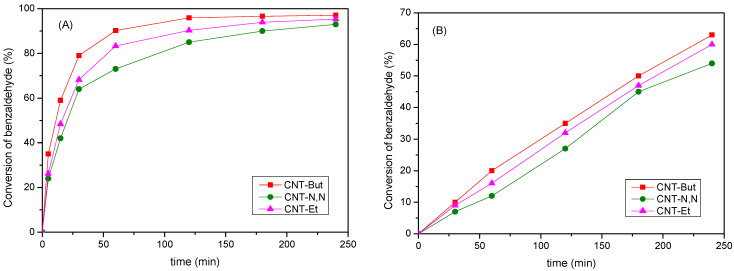
Conversion values for the Knoevenagel reaction between benzaldehyde and (**A**) ethyl cyanoacetate; (**B**) ethyl acetoacetate over CNT-A catalysts.

The conversion values of benzaldehyde (in wt.%) in the condensation with ethyl cyanoacetate obtained over CNT-A catalysts at 90 °C (Figure 8A) reveal that the reaction proceeded effectively when amines were anchored to the walls of nanotubes. On the contrary, the activity of CNT-O (not shown) was negligible. Therefore, the small number of metallic impurities that could remain in the oxidized nanotubes seemed to have no influence on the observed catalytic activity. Furthermore, as shown in Figure 6, the CNT-A samples contained small amounts of S, because the starting material for their synthesis, CNT-OCl, already contained it. However, the Cl/S atomic ratio (6.5) of this last sample (Figure 6) decreased significantly when treatment with amines occurred (with values of between 0.47 and 1.22). In addition, the atomic N/S ratios of the CNT-A samples were well above 1 (Figure 6). Therefore, the influence of the S on the catalytic activity seems to not be significant, although it cannot be completely ruled out.

The conversion of benzaldehyde for the three samples increased, as expected, with the reaction time. The reaction proceeded selectively and ethyl(2E)-2-cyano-3-phenyl-2-propenoate was the only reaction product obtained in all cases. Conversion values of between 93 and 97% were reached after 240 min, with the order of catalytic activity observed being CNT-N,N < CNT-Et < CNT-But. The best catalyst was CNT-But, which was the one showing the highest S_BET_ and V_pore_ values (Table 3). Considering that the catalyst with the greater amount of anchored amine was CNT-Et, it seems that for this reaction, not only the basicity of the nitrogen of the amine groups contributed to the activity, but also the textural properties of the samples, in agreement with other results reported for carbon nanotubes [30] and other materials [52,53] used as catalysts in Knoevenagel condensation. 

The same activity order was observed when the condensation of benzaldehyde was carried out with ethyl acetoacetate (pKa = 10.7) at 120 °C (Figure 8B). However, the conversion values were lower (54–63% at 240 min) than for ethyl cyanoacetate. Additionally, other secondary products were formed, along with the condensation product, due to different reactions, such as the decarboxylation of the Knoevenagel product, the aldolic condensations of the methylenic ester, and the oxidation of benzaldehyde, among others.

**Scheme 1:**
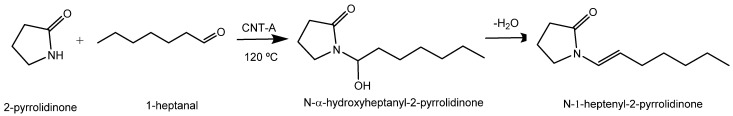
N-substitution of 2-pyrrolidinone with 1-heptanal over CNT-A catalysts.

Regarding the pKa of 2-pyrrolidinone (pKa = 11.3), which is close to that of ethyl acetoacetate (10.7), we considered that our catalysts may have been able to abstract the proton of 2-pyrrolidinone and to be active in the reaction between 1-heptanal and 2-pyrrolidinone to obtain the corresponding N-substituted-gamma-lactam (Scheme 1).

**Figure 9:**
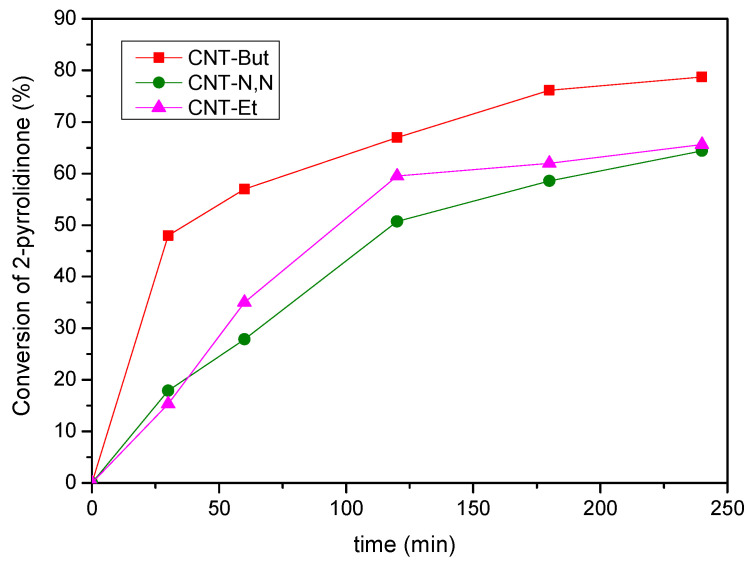
Conversion values for the reaction between 2-pyrrolidinone and 1-heptanal over CNT-A catalysts.

The catalytic activity was expressed in terms of the conversion of 2-pyrrolidinone, which was the limiting reactant. As deduced from Figure 9, the activity followed the same order as for the Knoevenagel condensations: CNT-N,N < CNT-Et < CNT-But. Conversion values of between 64.4 and 78.7% were achieved after 240 min. Again, the best catalyst was CNT-But, with the textural properties having an influence on the catalytic activity. This was followed by CNT-Et. Despite the fact this catalyst had lower S_BET_ and V_pore_ values than CNT-N,N, the content of mmol amine/g CNT for the first catalyst was the double that of the second one (Table 1), which indicates a combined effect of the basicity of nitrogen atoms and the textural properties of the catalyst over the final activity.

**Figure 10:**
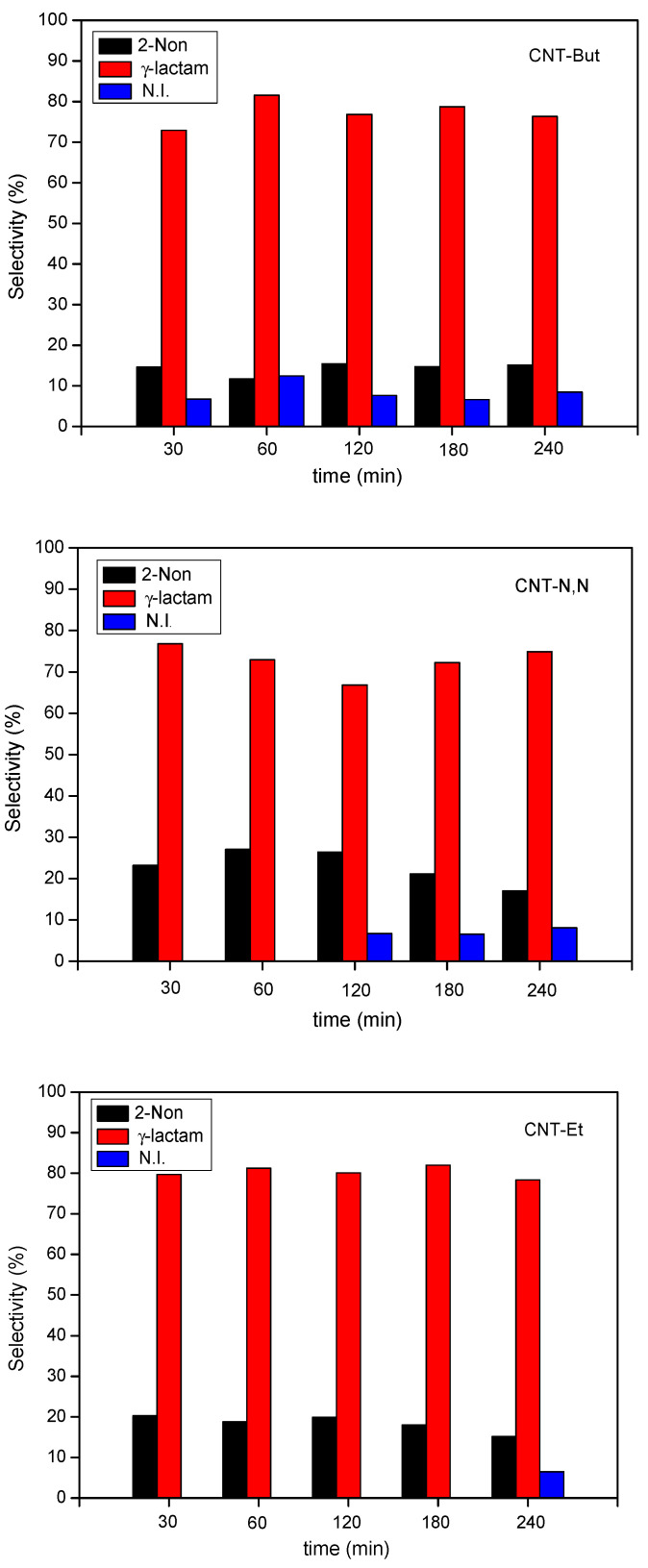
Selectivity values for the reaction between 2-pyrrolidinone and 1-heptanal over CNT-A catalysts.

The mass spectrum of the reaction products confirmed that N-substituted-gamma-lactam of type B, N-1-heptenyl-2-pyrrolidinone, was obtained (MS m/s: 182 (M+), 125, 97, 87, 70, 57, 42) (denoted as γ-lactam), together with 2-Nonenal, 2-pentyl- (denoted as 2-Non) (MS m/s: 210 (M^+^), 153, 139, 125, 109, 97), resulting from the auto condensation of 1-heptanal. Another minority non-identified product was also obtained (named as N.I.). The selectivity to the different products is displayed in Figure 10.

For CNT-Bu, the selectivity to γ-lactam was between 73 and 81%, with the highest value being observed at 30 min. From 120 min, the selectivity to the three formed products remained constant. In the case of the CNT-N,N sample, the selectivity values to γ-lactam (67–77%) were, in general, slightly lower than for the other two catalysts. The formation of N.I. product was detected from 120 min, and as a result, the selectivity to γ-lactam showed the lowest value at that time. For CNT-Et, the selectivity to γ-lactam remained between 78 and 82% and that for the 2-Non product was around 18–20% along the reaction time. The latter decreased slightly at 240 min because of the formation of the N.I. product.

**Figure 11:**
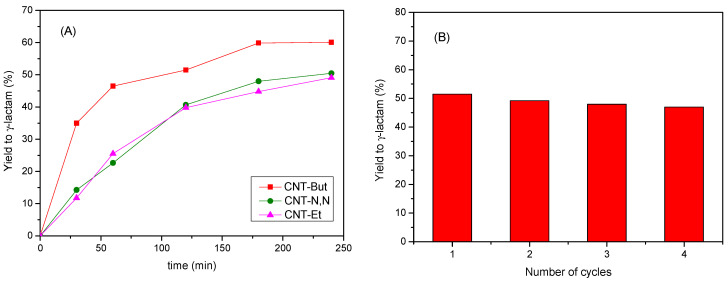
(**A**) Yield values to N-substituted-gamma-lactam for the reaction between 2-pyrrolidinone and 1-heptanal over CNT-A catalysts; (**B**) yield values to N-substituted-gamma-lactam obtained at 120 min over CNT-But in consecutive cycles.

The yields for the desired product, the N-substituted-gamma-lactam, are depicted in Figure 11A. For the best catalyst, CNT-But, a yield of 60% was achieved after 180 min. With the other two catalysts, the obtained yields were about 50% at 240 min.

The yield value to lactam obtained at 1 h with the CNT-But catalyst (47%) was very close to that reported by other authors (50%) in the same reaction [54], using active carbons impregnated with alkali metals as catalysts. However, the reaction conditions were different, since in our work the molar ratio 2-pyrrolidinone:1-heptanal was 1:3, while in their case, it was 1:3.5; therefore, it used a greater excess of aldehyde. Furthermore, the amount of catalyst used in the present work (3.8 wt.%) was significantly lower than that used (10 wt.%) in [54]. Therefore, considering that our reaction conditions imply less consumption of reagents and catalyst, it can be said that the CNT-A samples, and particularly CNT-But, are efficient catalysts in N-substituted-gamma-lactam synthesis. Additionally, as deduced from Figure 11B, this last catalyst could be used in four consecutive cycles, without a significant loss of activity, which shows the recyclability of the active sites.

## 4. Conclusions

In this work, we reported, for the first time, the synthesis of N-1-heptenyl-2-pryrrolidone via the N-substitution of 2-pyrrolidinone with 1-heptanal over carbon nanotubes functionalized with amino groups under free-solvent conditions. The catalysts were prepared by a process of acylation and subsequent amidation of pre-oxidized carbon nanotubes, and the incorporation of the nitrogen atoms was checked by different characterization techniques. The nature of the groups of the amine molecule was significant in terms of the functionalization degree, with this being the highest for ethylenediamine, for which the nitrogen atoms were more accessible and had no bulky substituents. The basic character of catalysts was firstly tested by Knoevenagel condensation of benzaldehyde with two different methylenic compounds. The order of the reaction, i.e., CNT-But > CNT-Et > CNT-N,N, was the same as that for the synthesis of N-1-heptenyl-2-pryrrolidone. The catalytic activity was influenced by a combined effect of the basicity, which depended on the amount of nitrogen, and the textural properties of solids. Yields of 45–60% to the desired product were achieved after 3 h. The recyclability of CNT-But catalyst was probed by checking the activity after four cycles of reaction. This process could be extended to the synthesis of other N-substituted γ-lactams, by selecting the starting γ-lactam and/or the type of aldehyde, which would result in a variety of compounds with interesting biological and pharmacological applications. Furthermore, the CNT-A samples were found to be active in Knoevenagel condensation, which is one of the imperative and essential condensation processes in synthetic organic chemistry. It would be expected that the use of these catalysts in the condensation of different aldehydes with several methylenic compounds could lead to the synthesis of a series of specialty chemicals and intermediates that have the potential to be widely used in various industries.

## Figures and Tables

**Table 1:** Elemental analysis and degrees of functionalization for CNT-A samples.

Sample	C (wt.%)	H (wt.%)	O (wt.%)	N (wt.%)	N/O *	mmol amine/g CNT
CNT-But	87.78	1.71	8.01	2.2	0.31	1.57
CNT-N,N	84.95	1.41	11.14	2.5	0.26	0.89
CNT-Et	78.47	1.92	14.37	5.24	0.41	1.87

* molar ratio.

**Table 2:** Textural properties and PCZ values for CNT, CNT-O, CNT-OCl, and CNT-A samples.

Sample	S_BET_ (m²/g)	V_pore_ (cm³/g)	V_meso_ (cm³/g)	V_micro_ (cm³/g)	D_meso_ (nm)	PCZ
CNT-O	259.9	0.832	0.902	0.101	13.4	3.4
CNT-OCl	280.0	1.026	1.675	0.107	21.8	n.d.
CNT-But	260.9	0.992	1.811	0.096	23.8	7.72
CNT-N,N	218.6	0.874	1.490	0.081	23.4	7.90
CNT-Et	192.9	0.801	1.333	0.070	21.5	7.80

S_BET_: specific surface area; V_pore_: pore volume at single point at P/P_0_ = 0.967; V_meso_: mesopore volume obtained by BJH between 2 and 50 nm; V_micro_: micropore volume obtained by Hortvath–Kawazoe method; D_meso_: average mesopore diameter (4 V/A) obtained by BJH; PZC: point of zero charge values.

**Table 3:** Weight loss determined by TGA in helium and degree of functionalization for CNT-A samples determined by TGA and EDX analysis.

Sample	Weight Loss (%)	mmol amine/g CNT (TG)	mmol amine/g CNT (EDX)
CNT-But	10.84	1.47	0.82
CNT-N,N	10.49	1.19	0.49
CNT-Et	17.5	3.0	1.03
